# World Endometriosis Research Foundation Endometriosis Phenome and Biobanking Harmonisation Project: I. Surgical phenotype data collection in endometriosis research

**DOI:** 10.1016/j.fertnstert.2014.07.709

**Published:** 2014-11

**Authors:** Christian M. Becker, Marc R. Laufer, Pamela Stratton, Lone Hummelshoj, Stacey A. Missmer, Krina T. Zondervan, G. David Adamson, WERF EPHect Working Group, G.D. Adamson, C. Allaire, R. Anchan, C.M. Becker, M.A. Bedaiwy, G.M. Buck Louis, C. Calhaz-Jorge, K. Chwalisz, T.M. D'Hooghe, A. Fassbender, T. Faustmann, A.T. Fazleabas, I. Flores, A. Forman, I. Fraser, L.C. Giudice, M. Gotte, P. Gregersen, S.-W. Guo, T. Harada, D. Hartwell, A.W. Horne, M.L. Hull, L. Hummelshoj, M.G. Ibrahim, L. Kiesel, M.R. Laufer, K. Machens, S. Mechsner, S.A. Missmer, G.W. Montgomery, A. Nap, M. Nyegaard, K.G. Osteen, C.A. Petta, N. Rahmioglu, S.P. Renner, J. Riedlinger, S. Roehrich, P.A. Rogers, L. Rombauts, A. Salumets, E. Saridogan, T. Seckin, P. Stratton, K.L. Sharpe-Timms, S. Tworoger, P. Vigano, K. Vincent, A.F. Vitonis, U.-H. Wienhues-Thelen, P.P. Yeung, P. Yong, K.T. Zondervan

**Affiliations:** a Nuffield Department of Obstetrics and Gynaecology, University of Oxford, Oxford, United Kingdom; b Endometriosis CaRe Centre Oxford, University of Oxford, Oxford, United Kingdom; c Division of Gynecology, Department of Surgery, Boston Children's Hospital and Harvard Medical School, Boston, Massachusetts; d Department of Obstetrics, Gynecology, and Reproductive Biology, Brigham and Women's Hospital and Harvard Medical School, Boston, Massachusetts; e Boston Center for Endometriosis, Boston Children's Hospital and Brigham and Women's Hospital, Boston, Massachusetts; f Program in Reproductive and Adult Endocrinology, Intramural Program, *Eunice Kennedy Shriver* National Institute of Child Health and Human Development, National Institutes of Health, Bethesda, Maryland; g World Endometriosis Research Foundation, London, United Kingdom; h Channing Division of Network Medicine, Department of Medicine, Brigham and Women's Hospital and Harvard Medical School, Boston, Massachusetts; i Department of Epidemiology, Harvard School of Public Health, Boston, Massachusetts; j Wellcome Trust Centre for Human Genetics, University of Oxford, Oxford, United Kingdom; k Palo Alto Medical Foundation Fertility Physicians of Northern California, Palo Alto, California

**Keywords:** Endometriosis, standardization, harmonization, phenotyping, laparoscopy, EPHect SSF

## Abstract

**Objective:**

To standardize the recording of surgical phenotypic information on endometriosis and related sample collections obtained at laparoscopy, allowing large-scale collaborative research into the condition.

**Design:**

An international collaboration involving 34 clinical/academic centers and three industry collaborators from 16 countries.

**Setting:**

Two workshops were conducted in 2013, bringing together 54 clinical, academic, and industry leaders in endometriosis research and management worldwide.

**Patient(s):**

None.

**Intervention(s):**

A postsurgical scoring sheet containing general and gynecological patient and procedural information, extent of disease, the location and type of endometriotic lesion, and any other findings was developed during several rounds of review. Comments and any systematic surgical data collection tools used in the reviewers' centers were incorporated.

**Main Outcome Measure(s):**

The development of a standard recommended (SSF) and minimum required (MSF) form to collect data on the surgical phenotype of endometriosis.

**Result(s):**

SSF and MSF include detailed descriptions of lesions, modes of procedures and sample collection, comorbidities, and potential residual disease at the end of surgery, along with previously published instruments such as the revised American Society for Reproductive Medicine and Endometriosis Fertility Index classification tools for comparison and validation.

**Conclusion(s):**

This is the first multicenter, international collaboration between academic centers and industry addressing standardization of phenotypic data collection for a specific disease. The Endometriosis Phenome and Biobanking Harmonisation Project SSF and MSF are essential tools to increase our understanding of the pathogenesis of endometriosis by allowing large-scale collaborative research into the condition.

**Discuss:** You can discuss this article with its authors and with other ASRM members at **http://fertstertforum.com/beckerc-werf-ephect-i/**

Since the first publication on endometriosis by von Rokitansky more than 150 years ago (1), many uncertainties remain about the diverse clinical, molecular, and societal aspects of the disease. It is generally accepted that endometriosis is a heterogeneous disease with respect to its natural history, disease burden, extent of inflammation, state of progression, and phenotypic presentation of lesions and symptoms. Widely used classification systems such as the one developed and revised by the former American Fertility Society (AFS; now the American Society for Reproductive Medicine, ASRM) have not been helpful in phenotyping the disease (2), discerning patient morbidity, or predicting treatment response or prognosis.

It is now well established that no correlation exists between revised AFS stage or other classification systems and the presentation or severity of pain symptoms 3, 4. The Endometriosis Fertility Index (EFI) has been validated to predict clinical fertility outcome but is not designed to correlate with other clinical symptoms such as pain (5). Consequently, it is challenging to correlate degree of symptoms with disease severity or establish an accurate prevalence of clinically significant disease based solely on symptomatology 6, 7 so that phenome-specific diagnostic and treatment discovery can advance.

Although phenotypic categorization based on lesion appearance and location has been suggested 8, 9, no systematic validation of these mutually exclusive categories or longitudinal studies of their role in treatment response and disease prognosis exist. Furthermore, molecular and genetic marker discernment has not yet been applied sufficiently to reinforce or refute these gross phenotypic groupings. Indeed, it is also not clear how to categorize/treat a patient as a “whole” woman within whom all or some of these lesion subtypes may exist. It must always be acknowledged that, given current requirements for surgical diagnosis and intervention, the phenotype observed and biologic samples collected at surgery are only reflective of that specific point in time and may not be evidence of or even correlated with the disease phenotype across the life course.

Furthermore, it is unclear what biologic or phenomic characteristics of endometriosis, or other possible parameters, are predictive of progression of the disease 10, 11. Is it simply an increase in lesion burden, suggested by a change in color, texture, volume, or location of endometriotic lesions, or does progression involve a shift in, for example, gene/protein expression profiles or in immune response—or a combination of both—that may be measured using either localized or systemic biologic specimens? It remains to be determined whether such parameters are etiologic or a consequence of the disease or of disease-related symptoms.

To date, no non- or minimally invasive diagnostic test exists to aid the diagnosis of endometriosis, elucidate the natural history of the disease, or predict treatment efficacy related to lesions or symptoms 12, 13, 14. Biomarker studies have shown varying and often conflicting results. Currently available data sets on endometriosis cases and controls typically: [1] lack surgical phenotypic and symptomatic detail combined with biological sample information; [2] are insufficiently consistent in terms of the type of data collected and protocols used to allow the collaborative exploration of the above-mentioned associations; or [3] are too small to have sufficient power. While there is consensus that laparoscopy remains the gold standard for a definitive diagnosis of endometriosis 15, 16, 17, we suggest taking full advantage of the diagnostic aspect of the procedure by collecting standardized detailed information at the time of surgery and thus optimizing the characterization of the surgical phenotype both for clinical and research purposes. To date, even in well-established and recognized endometriosis clinical research centers, no consensus exists on even the minimum surgical information that should be collected to perform clinical and basic science studies.

The World Endometriosis Research Foundation (WERF) Endometriosis Phenome and Biobanking Harmonisation Project (EPHect) is a global initiative involving 34 clinical/academic and three industrial collaborators from 16 countries, with the mission to develop a consensus on standardization and harmonization of phenotypic surgical/clinical data and biological sample collection methods in endometriosis research. Specifically, to facilitate large-scale internationally collaborative, longitudinal, epidemiologically robust, translational, biomarker and treatment target discovery research in endometriosis, EPHect provides evidence-based guidelines on: [1] detailed surgical, clinical, and epidemiological phenotyping (phenome) data to be collected from women with and without endometriosis to allow collaborative subphenotype discovery and validation analyses; and [2] standard operating procedures (SOPs) for collection, processing, and long-term storage of biological samples from women with and without endometriosis.

To achieve these goals, WERF conducted two workshops in March and July 2013, bringing together international leaders in endometriosis research to develop and reach consensus on evidence-based EPHect phenome collection and SOP guidelines. Draft consensus questionnaires and SOPs were subsequently reviewed during several rounds of expert review by the WERF EPHect Working Group. To the best of our knowledge, this harmonization initiative is unique in terms of its scope—addressing standardization of phenotypic data collection and biological sampling protocols simultaneously for a specific disease—with consensus reached from a large number of academic as well as industrial leaders in endometriosis research. It also is a direct answer to the key priority of phenome data collection and SOP harmonization identified in Endometriosis Research Directions workshops held in 2008 (18) and 2011 (19) and will allow the investigation of a substantial number of other research priorities highlighted.

It is important to note that EPHect does not aim to evaluate any past, present, or future research nor will it instruct surgeons on how to perform their procedures. The recommendations presented here are primarily aimed at improving and facilitating research and are not necessarily applicable to the clinical management of endometriosis. Rather, EPHect aims at a significant improvement in the comparability of endometriosis studies and enhanced opportunities for collaborative research with much improved statistical power—particularly for less prevalent but nonetheless informative disease (sub-) phenotypes. By increasing the understanding of endometriosis, EPHect hopes to reduce the costs of health care and clinical studies of endometriosis, ultimately leading to a more individualized treatment approach and a consequent improvement of the lives of many millions of women and their partners.

Here we report the EPHect consensus on standard recommended surgical data and sample collection in endometriosis research (part I). Three companion papers in the series cover the other EPHect endpoints: standardized collection of nonsurgical/clinical and epidemiological phenomic data through patient-administered questionnaires (part II) (20); SOPs for biological fluid (part III) (21); and tissue (part IV) (22) sample collection, processing, and long-term storage to enable cellular, genetic, and molecular, proteomic, metabolomics, metabonomic, and transcriptomic phenome studies. The data collection tools and SOPs will be updated regularly, based on: [1] feedback from centers adopting the current instruments and protocols; [2] protocols generated for new sample types; and [3] a 1-year, and thereafter triannual, review of literature and other publicly available evidence and will be freely accessible through the WERF EPHect website (endometriosisfoundation.org/ephect). It is anticipated that the EPHect initiative will lead to an unprecedented standardization of integrated phenomic data and biological specimen collection to enable large-scale collaborative research into this highly heterogeneous and still enigmatic disease.

## Materials and methods

**Figure 1:**
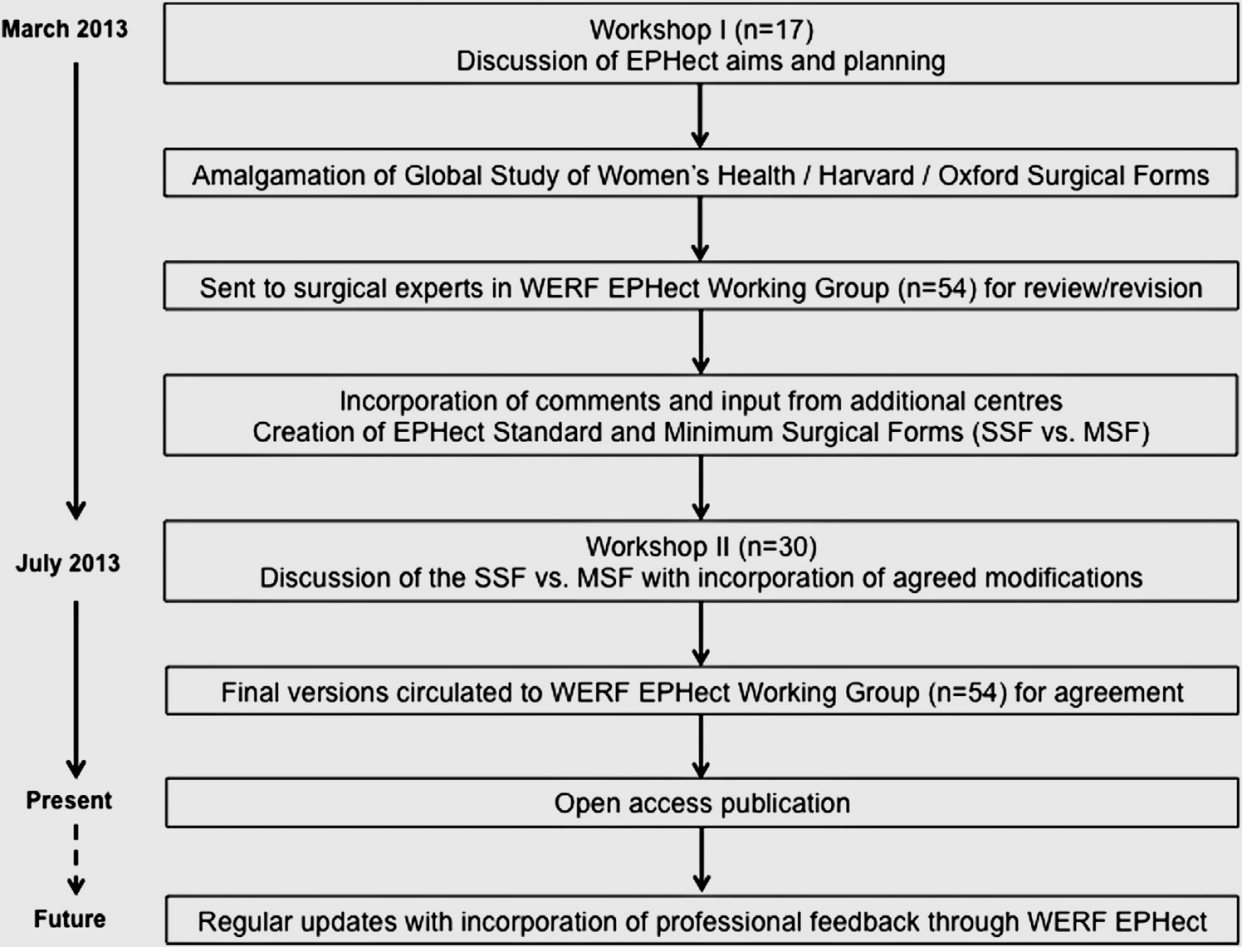
Flow diagram depicting the WERF EPHect development and consensus process (surgical data collection).

We conducted two workshops in March and July 2013, bringing together leaders in endometriosis research worldwide to develop and reach consensus on evidence-based phenome collection and SOP guidelines, followed by several rounds of expert review by the WERF EPHect Working Group (Fig. 1). During Workshop I, four areas of standardization and harmonization were defined: (I) surgical phenotyping; (II) nonsurgical clinical/epidemiologic phenotyping; (III) fluid sample; and (IV) tissue sample collection, processing, and storage protocols for molecular and genetic phenotyping. To date, the WERF EPHect global initiative has involved 34 clinical/academic centers and three industry collaborators (54 participants) from 16 countries on five continents.

The development of the EPHect surgical data collection instrument was initially based upon a postsurgical scoring sheet originally developed as part of the WERF-funded Global Study of Women's Health 23, 24, which had recently been extended and piloted in a collaboration between the Boston Center for Endometriosis and the Endometriosis CaRe Centre Oxford. The scoring sheet—containing general and gynecological information about the patient, the procedure, extent of disease, and the location and type of endometriotic lesion, as well as any other findings, along with existing tools for disease classification such as the revised AFS (2) and EFI (25)—was discussed and extended during several review rounds by experts in the field of endometriosis using surgical data collection tools that were in use at their centers (Fig. 1).

While validity, reliability, and scientific advancement in endometriosis research are the main goals of EPHect, an important point acknowledged by the EPHect Working Group was that there are likely to be differences in resources and logistics among centers that influence feasibility of adherence to some of the strictest standards of data collection and SOP implementation. EPHect therefore agreed on two tiers for all data collection instruments as well as for biological sample SOPs: standard recommended and minimum required. Standard surgical data were considered of central importance for current and future advancement in understanding the biology of disease and investigation of the effects of treatment on symptoms and disease recurrence. The minimum required version included data collected as a basic requirement for more limited research studies in settings where completion of the standard instrument is logistically impossible. The two versions resulting from this consultation were presented at Workshop II. Of 54 invitees, 30 attended either in person or via video link. During Workshop II, the standard recommended and the subset for minimum required collections were agreed upon, based on a list of individual, local, national, or international circumstances (Table 1). Notes regarding comments and discussions among participants were taken during the meeting and incorporated into the final versions. Both forms are designed for surgery involving women with confirmed endometriosis and symptomatic or asymptomatic controls/comparison women (e.g., women undergoing laparoscopic sterilization or those evaluated for symptoms but found not to have endometriosis).

**Table 1:** Individual, local, national, and international factors that may influence the extent and type of data collected.

Factor
Financial situation Local organizational structure Cultural differences Research question Knowledge of staff Availability of time Motivation of surgeon or institution Ethics approval by the local Institutional Review Board

Approval by an Ethics Committee or Institute Review Board was not required for formation of the EPHect Working Group, review of existing literature, or consensus regarding best practices for endometriosis research described within the WERF EPHect four manuscript series. This endeavor did not include data from human subjects. A comprehensive list of declared conflicts of interest for each of the authors and members of the EPHect Working Group is provided.

## Results

During the EPHect consultation process, it became apparent that there was little or no consistency in the surgical endometriosis data collected by different centers, both in terms of the extent of information gathered and the topics, definitions, or specifications used. The development of the two EPHect surgical data collection forms, the standard (recommended) surgical form (EPHect SSF) and minimum (required) surgical form (EPHect MSF), is described below. All recommendations are of a purely scientific nature and not meant as clinical guidance. To complement these data we strongly recommend using the EPHect patient questionnaires covering important aspects of disease symptomatology (EPQ-S and EPQ-M), which were developed and agreed upon by the same group of experts (20).

### EPHect SSF

The EPHect SSF was developed with the aim to collect all currently deemed relevant and important information describing the visual endometriosis phenotype and surgical treatment that would allow clinically and scientifically meaningful studies (Supplemental Appendix 1) (19). The EPHect SSF is divided into two parts. The first part asks for detailed information about clinical covariates: the current menstrual cycle, current hormone treatment, and history of previous endometriosis surgery, as well as any imaging findings before the procedure. The second part concentrates on intraoperative findings including the type and duration of the procedure; and the extent, exact location, and color of endometriotic lesions, with a particular focus on size of endometrioma and endometriotic nodules. It allows for an exact description of tissue biopsies (see section on biological sample collection), including their location and appearance, and surgical treatment of lesions. For guidance purposes we have added pictures of representative endometriotic lesions (Supplemental Appendix 2). From experience there can be variability in how such pictures are interpreted, for example, in terms of lesion color or depth of invasion. This is usually not driven by screen differences but by actual heterogeneity in surgeon color assignment within the operating theater or by the extent of surgical exploration and/or treatment. The occurrence and description of any intraoperative complications is also documented, as well as the extent of residual endometriosis after surgery and a detailed description of any other pathological findings during the procedure (e.g., uterine fibroids, scarring, adhesions). Pilot work in several centers has shown that—after an initial brief learning period—the EPHect SSF typically requires 1–3 minutes to complete, depending on the extent of disease and sample taking to be recorded. This time can be reduced if the patient history (part 1) is completed in advance by a clinical or research team member.

### EPHect MSF

We recommend including as many of the surgical data items included in the EPHect SSF as possible, to allow for maximization of collaborative work with other EPHect research centers around the world. The sole aim of developing the EPHect MSF was to identify the essential, basic, surgical information that a surgeon under considerable time constraints, based at a center without research support, would be able to complete accurately and consistently immediately after surgery (Supplemental Appendix 3). The EPHect MSF would enable a group to start gathering potentially relevant surgical phenotypic information where such information was not systematically collected before. Similar to the EPHect SSF, the EPHect MSF is also divided into two parts, asking about clinical covariates and intraoperative findings, respectively, but in less detail. While during EPHect Workshop II it was agreed that menstrual history and knowledge about hormone use is a required minimum and should be standard knowledge for any surgeon performing a surgical gynecological procedure, information on previous surgeries was not deemed to be part of the minimum required form. Moreover, the EPHect MSF documents the type and duration of the surgical procedure, but the extent and exact location of endometriosis lesions and other intraoperative findings are less detailed than recorded in the EPHect SSF.

### Video/Photo Documentation

To evaluate the presence or absence of endometriotic lesions, adhesions, and cysts, it is crucial that the entire pelvis and abdominal cavity be systematically and meticulously searched with a laparoscope. As endometriosis lesions can be small, colorless, or vesicular, it is critical that the peritoneal areas are viewed from close proximity 26, 27. A “close tip” technique is recommended (2–5 cm distance between laparoscope and peritoneal surface) so that the laparoscopic magnification can be used to assist in visualization of lesions. It is important to include evaluation of the posterior surface of both ovaries. However, limited handling of the peritoneum is essential as small peritoneal petechiae are likely to form especially in an inflamed environment typical for endometriosis (28).

Access may be limited owing to the extent of disease or comorbidities, such as adhesions, but every effort should be made to inspect all possible locations. These include the anterior and posterior cul-de-sac, all surfaces of the pelvic organs, the pelvic sidewalls, and the mid and upper abdomen.

**Figure 2:**
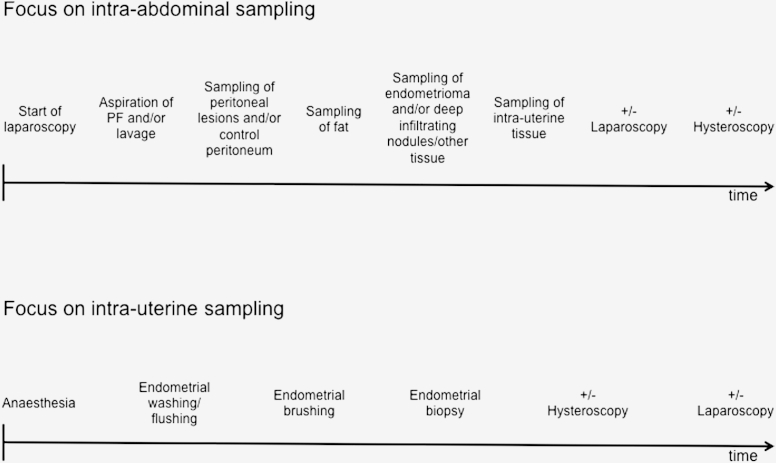
Schematic distribution of video and photo imaging encompassing the entire female pelvis and its organs. Each zone is recommended to be photographed separately.

Where permitted and feasible, video recording of pelvic explorations and the actual surgical procedure should be considered (29). In addition, photo documentation is strongly encouraged and considered to be the standard recommended for research purposes, which will provide an objective record of the reported data. Also, it should be considered that while it is understood that there is interest in exploring the gross and molecular phenotype of individual lesions, it may be that unique and critical information can be discovered from the colony/cluster/microenvironment of lesions proximal to each other. These phenotypic details can only be documented and quantified from video and/or photographic evidence. Figure 2 shows the photo documentation to be collected as the standard recommended by the EPHect Working Group. The pelvis is divided into six zones, two in the midline and two for each side (Table 2). To capture each zone, a distance of 5–10 cm of the laparoscope to the peritoneal surface will be required. As most endometriotic disease is found in the pelvis, this distribution will include most of endometriotic lesions. If very small lesions are present, then it is important to capture them, if necessary with an additional, close-range picture 26, 30. Efforts should be made to photo and/or video document any other endometriotic sites outside the pelvis. A photograph of the pelvic cavity at the end of surgery, with particular focus on capturing any residual disease, should be included in the standard visual documentation.

**Table 2:** Suggested zones for surgical video and photographic documentation.

Zone	Boundaries	Contents
I	Bilateral: round ligaments Posterior: uterus Midline anterior abdominal cavity limited by the round ligaments bilaterally and the uterus posteriorly	Midline anterior abdominal peritoneum Anterior surface of the broad ligaments Bladder dome Internal ring and inferior epigastric vessels
II	Anterior: bladder peritoneum Bilateral: broad ligaments and adnexae Caudal: torus uterinus	Uterus
III	Anterior: torus uterinus Bilateral: uterosacral ligaments Posterior: sacral pelvic brim	Pouch-of-Douglas/posterior cul-de-sac Rectovaginal septum Sigmoid colon Presacral peritoneum
IV	Anterior: right round ligament Medial: right broad ligament and adnexe	Right lateral peritoneum Anterior surface of right broad ligament
V	Medial: uterus and right uterosacral ligament Anterior: right fallopian tube Lateral: right infundibulopelvic ligament	Fallopian tube and ovary Mesovarium Posterior surface of the broad ligament Ovarian fossa Vessels and ureter
VI	Anterior: right round ligament Medial: right broad ligament and adnexe	Left lateral peritoneum Anterior surface of left broad ligament
VII	Medial: uterus and left uterosacral ligament Anterior: left fallopian tube Lateral: left infundibulopelvic ligament	Fallopian tube and ovary Mesovarium Posterior surface of the broad ligament Ovarian fossa Vessels and ureter
VIII	Not applicable	Other abdominopelvic areas including: Adipose tissue Bowel Ureter Anterior abdominal wall Diaphragm
IX	Not applicable	Other sites outside the abdominopelvic area including: umbilicus, scars, chest, etc.

As the era of digital photography and electronic medical records evolves, this is a highly appropriate time to innovate the methods by which surgical findings are documented (31). Implementation of a standardized systematic approach for laparoscopic pelvic examination as suggested here by EPHect and recommended by others 16, 32 will enhance diagnostic accuracy, help diagnose lesions in anatomically challenging locations, and provide a novel level of standardization that is important for both clinical patient-focused and academic research aims.

### Biological Sample Collection

Potential biological samples relevant to endometriosis research that could be collected during laparoscopic surgery include, but are not limited to, endometriotic disease. Detailed EPHect SOPs for the collection, processing, and long-term storage of such samples, and evidence upon which these are based, are provided in two companion papers 21, 22.

We recommend that samples be collected sequentially according to a prespecified, well-documented, protocol. For any samples collected intraoperatively, possible sources of contamination include fluids such as blood or distension fluids, for example, from hysteroscopy, general or prolonged exposure of the peritoneal surfaces to dry and cold CO_2_, anesthetic and premedication or other drugs, and exogenous exposures 33, 34, 35, 36. The length of time between surgical excision/extraction of the samples and subsequent processing/storage, along with the conditions in which samples are kept during this period, can have a very substantial impact on the molecular profiles that are detailed in the companion EPHect papers 21, 22. It is essential therefore that optimal SOPs be implemented from the moment of surgical extraction of the sample, allowing the highest quality data to be obtained from their analysis. Recording of metadata such as the times of the start of a procedure and of sampling are important to document 21, 22.

**Figure 3:**
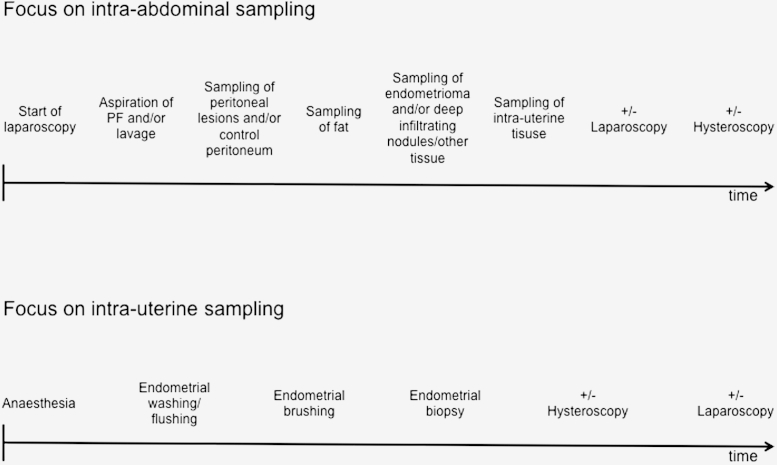
Suggested timeline for biological sample collection depending on research question.

In general, sampling should be performed as early as possible to diminish a possible impact of anesthetic drugs and the above-mentioned confounding variables on results (37). Provided it is clinically justifiable, the order of sampling is dictated by the research question (Fig. 3). For example, if intra-abdominal sampling (peritoneum, peritoneal fluid, endometriotic disease) is the main focus, it is recommended to perform laparoscopy before hysteroscopy to avoid contamination from hysteroscopic fluid. However, it may be clinically indicated to start the operation with the hysteroscopy. For research purposes, the order of surgical procedures and the type of the hysteroscopic fluid should be recorded as indicated on the EPHect SSF.

If peritoneal fluid is collected for research purposes, this should be the first intra-abdominal sample collected to reduce the risk of contamination with blood, cyst fluid, or tissue. The volume of peritoneal fluid is influenced by the ovaries and the menstrual cycle (38). If no or very limited amounts of peritoneal fluid are available, then a lavage with physiologic saline (10 mL) over the pelvic organs and walls is the standard volume and site of lavage recommended. Ideally, any visible peritoneal fluid should be collected and stored separately before lavage. Higher volumes of lavage fluid should be avoided since peptides and secreted factors withstand long-term storage and future analysis better when concentrated (Raymond Anchan, M.D., personal communication). The optimal position to collect either peritoneal or lavage fluid is in a 30° reverse Trendelenburg position (39).

Next, endometriotic peritoneal lesions, and—if present—adhesive sites should be collected. This is also the point of surgery at which “normal”/control peritoneal tissue should be collected if approved as part of the research protocol by the center's human subjects research approval process. Collection and evaluation of control/normal adjacent tissue is a standard method within many fields, including cancer research, as it offers an extensive array of within-woman comparative molecular and genetic testing (40) and should be included when possible to advance endometriosis research. However, again, the order of sample collection may change depending on the research question. Owing to the anatomic location and possible surgical complexity, endometriomas and deep infiltrating nodules are commonly the last samples to be collected. Sometimes large endometriomas may need to be mobilized or removed to gain access to the posterior cul-de-sac/Pouch of Douglas. As per the standard recommendation, for any abdominal sample, the temperature of the CO_2_ entering the abdomen and the presence or absence of a gas humidifier should be recorded for each sample (41).

If the main research focus is on uterine sampling (eutopic endometrium and myometrium), then it may be preferable to begin with the endometrial biopsy to reduce the potential direct or indirect effect of anesthetic drugs or potential endocrine or paracrine influences on the samples (Fig. 3). In any scenario, it is recommended as standard to collect endometrial samples before the insertion of a uterine manipulator, as this is likely to alter sample quality. Evidence exists that endometrial injury improves embryo implantation in the subsequent conception-attempting cycle (42), indicating that such injury can result in changes in the endometrial phenotype. Intrauterine procedures such as hysteroscopy or endometrial biopsy should therefore be avoided for at least one full menstrual cycle before collection of samples for evaluation of endometriosis-associated changes in the endometrial phenotype. Alternatively, the type and date of any prior intrauterine procedure should be recorded as part of the minimum required form.

If surgically feasible, the use of electric or light energy should be avoided for all tissue collections, as electrosurgery/cautery artifacts may impact on the histological interpretation of the tissue (43) and possibly on the expression of biomarkers. The extent of this effect is dependent upon factors such the type of device (laser or plasma jet versus mono- or bipolar electrosurgery) and the strength and time of energy used. If thermal energy is necessary, then it is recommended to use laser or plasma jet and as little energy as clinically possible and to leave a safety margin of 5 mm.

## Discussion

Our knowledge of endometriosis and its associated symptoms is still in its infancy with regard to its etiology, natural history, noninvasive diagnostic methods, and optimal treatment modalities, despite years of basic, observational, and experimental (clinical trial) research. Many studies that were aimed at identifying sensitive and specific diagnostic markers for endometriosis have been underpowered, had various methodological flaws, or had uninterpretable or contradictory results (14). While some studies have identified potentially interesting biomarker candidates, other studies could not replicate the findings, thus questioning their validity and reliability. Undoubtedly, differences in study design, execution, and analysis may be responsible for these discrepancies, but an important reason is also likely to be the lack of standardized phenotypic definition and protocol-based sample collection across studies, generating substantial study-related variability in results. Detailed, standardized, surgical characterization of endometriosis is a vital starting point to allow more homogeneous subtypes to be defined in studies and for molecular profiling and biomarker results to be correlated with disease entities.

As the opportunity for large-scale molecular profiling of tissues from women with endometriosis advances—with ever increasing sensitivity and robustness of technologies at ever decreasing costs—the need for case and control/comparison data sets with available biological samples relevant to the disease, and collected using standard protocols, is obvious. However, it is impossible to achieve these goals without detailed phenotyping during surgical observations and from clinical symptomatology and epidemiologically relevant covariates. Standardization of such phenotyping will allow, for the first time, definition of endometriosis patient subgroups to be considered systematically, across phenotypically similar data sets, providing a real opportunity for studies to be powered sufficiently to address research questions specific to such subgroups. Indeed, the lack of surgical characterization of patient subgroups has been a major concern in clinical trials of novel treatment approaches in endometriosis, many of which have been unsuccessful or have remained unreported 44, 45. One of the central criticisms of these studies is that either insufficient or irrelevant information was collected from patients, and therefore patient description or selection was suboptimal. Thus, to enable the multicenter collaborations envisaged by the WERF EPHect initiative, it is essential that centers adopting the WERF EPHect instruments and SOPs ensure that patients provide informed consent that allows their data and biological samples to be used in future multicenter (inter)national collaborations and that appropriate Ethics Committee and Institute Review Board approval is obtained that allows for such collaborations.

One central aim of WERF EPHect—in answer to research priorities identified in endometriosis 18, 19—is to standardize phenotypic data collection across studies of endometriosis, to allow large-scale research using multiple data sets that are characterized using the same phenotypic definitions as well as to allow replication of results between data sets. The surgical data collection tools presented here, in combination with the EPHect nonsurgical data collection tools (20), constitute the consensus of leading clinical, epidemiological, and/or basic science experts in the field of endometriosis and were based on extensive current knowledge and anticipated future relevance. While it is possible that some of the information about patient history and intraoperative findings collected in the EPHect instruments will turn out to be clinically irrelevant, it is vital that these data are collected and processed until a decision about their importance and significance can be explored. Also, as scientific foci shift, novel research fields, and methods emerge, and knowledge of endometriosis accumulates, it is likely that phenotypic data that may seem irrelevant at the moment may become important in the future for different, specific, research questions on patient subtypes.

We strongly advise the EPHect SSF, rather than EPHect MSF, be adopted where possible, as this will allow the generation of well-characterized phenotypic data sets that will be most versatile in their application to current and future research questions in the field of endometriosis. The evidence base for all EPHect data collection instruments and SOPs will be reviewed continuously upon feedback provided by investigators, and through systematic surveys and follow-up reviews after 1 year and every 3 years thereafter. Thus, investigators are strongly encouraged to provide such feedback. Updates of instruments will remain freely accessible to the research community through the WERF EPHect website (endometriosisfoundation.org/ephect). We ask that publication of results that are generated using WERF EPHect data and sample collection protocols appropriately reference the sources, including version numbers, of the instruments used. In the next phase of the EPHect initiative, WERF aims to: [1] develop freely available stand-alone applications as well as web-based systems to facilitate center-restricted data entry and reduce costs and time expenditure to individual centers; and [2] amalgamate a voluntary registry of centers using EPHect data collection tools and biological sample SOPs that would offer any investigator a transparent platform for the establishment of new collaborations.

We hope that the recommendations we have presented on surgical phenotyping, in combination with the other data collection and sample protocols described in our EPHect companion manuscripts 20, 21, 22, will inspire a new chapter of globally standardized data and sample collection in endometriosis research, foster many new collaborations among existing centers, and encourage other endometriosis patient centers that have not yet embarked on research to join. This will surely aid our quest to improve of the quality of life of millions of women affected by endometriosis, and their partners, worldwide.

## Supplementary data

Supplemental Appendix 1 Standard (recommended) surgical form (EPHect SSF).

Supplemental Appendix 2 Minimum (required) surgical form (EPHect MSF).

Supplemental Appendix 3 Example pictures of endometriotic lesions.
