# Erratum

**DOI:** 10.1016/j.fertnstert.2015.07.1167

**Published:** 2015-10

The article by Becker et al., “World Endometriosis Research Foundation Endometriosis Phenome and Biobanking Harmonisation Project (EPHect): I. Surgical phenotype data collection in endometriosis research” (Fertil Steril 2014;102:1213–22), contains two errors in the Results section.

On page 1217, under the heading “Video/Photo Documentation,” the sentence in the third paragraph should read, “The pelvis is divided into seven zones, three in the midline and two lateral for each side (Table 2).”

On page 1219, in Table 2, under the column “Boundaries,” Zone VI should read, “left round ligament and left broad ligament” instead of right.

The authors apologize for the errors.

