# Supplementary material for: World Endometriosis Research Foundation Endometriosis Phenome and Biobanking Harmonisation Project: I. Surgical phenotype data collection in endometriosis research
Source: Fertil Steril. 2014 Nov;102(5):1213–22. doi: 10.1016/j.fertnstert.2014.07.709 (PMC4230690; doi:10.1016/j.fertnstert.2014.07.709)
Supplement: Supplemental Appendix 1 — Standard (recommended) surgical form (EPHect SSF). [file mmc1.docx]

Surgeon ID: ________________________ Patient ID: ________________________ Date: __ __ /__ __ /__ __ __ __

DD MM YYYY

**I. Menses:** LMP: __ __ /__ __ /__ __ __ __ Cycle day: __ __ Currently bleeding? **🞏** **No** **🞏** **Yes**

DD MM YYYY

**II. Current hormonal treatment: 🞏** **No** **🞏** Do not know **🞏** **Yes**

**🞏** COCP **🞏** POP **🞏** Depot progestin

**🞏** GnRH agonist **🞏** GnRH antagonist **🞏** IUCD

**🞏** Other ___________________

Last application: __ __ /__ __ /__ __ __ __

DD MM YYYY

**III. Previous surgical diagnosis of endometriosis:** **🞏** **No** **🞏** Do not know **🞏** **Yes**

If Yes: 1) Hospital? ____________________ When? __ __ /__ __ /__ __ __ __ Procedure(s)? ___________ ____________

DD MM YYYY

2) Hospital? ____________________ When? __ __ /__ __ /__ __ __ __ Procedure(s)? ___________ ____________

DD MM YYYY

3) Hospital? ____________________ When? __ __ /__ __ /__ __ __ __ Procedure(s)? ___________ ____________

DD MM YYYY

**IV. Imaging prior to surgery: 🞏** **No** **🞏** **Yes**

**🞏** Ultrasound Dates: __ __ /__ __ /__ __ __ __

DD MM YYYY

**🞏** MRI Dates: __ __ /__ __ /__ __ __ __

DD MM YYYY

Findings:

**🞏** Cyst(s) left size: 1. __ __cm 2. __ __cm 3. __ __cm

**🞏** Cyst(s) right size: 1. __ __cm 2. __ __cm 3. __ __cm **🞏** Rectovaginal nodule

**🞏** Bladder nodule

**🞏** Ureter involvement

**🞏** Left

**🞏** Right

**🞏** Uterine anomalies

**🞏** Fibroids

**🞏** Polyps

**🞏** Adenomyosis

**🞏** Other: ____________________

**V.** **Procedures:** Total surgical time: __ __ __ min.

Uterine cavity surgery Hysteroscopy before laparoscopy: 🞏 **No** 🞏 **Yes**

Hysteroscopy after laparoscopy: 🞏 **No** 🞏 **Yes**

Findings: 🞏 Normal

🞏 Abnormal: ___________ __________

🞏 Diagnostic Hysteroscopy

🞏 Polypectomy

🞏 Resection of fibroid

🞏 Resection of endometrium

🞏 Resection of septum/adhesions

🞏 Other ____________________

Ovarian surgery Surface: 🞏 Excision 🞏 Left 🞏 Right 🞏 Both: 🞏 Laser 🞏 Monopolar 🞏 Bipolar

🞏 Ablation 🞏 Left 🞏 Right 🞏 Both: 🞏 Laser 🞏 Monopolar 🞏 Bipolar

🞏 Fulguration 🞏 Left 🞏 Right 🞏 Both

🞏 Ovariolysis 🞏 Left 🞏 Right 🞏 Both

🞏 Temporary suspension 🞏 Left 🞏 Right 🞏 Both

🞏 Oophorectomy 🞏 Left 🞏 Right 🞏 Both

🞏 Ovarian cystectomy 🞏 Left 🞏 Right 🞏 Both

🞏 Ovarian reconstruction 🞏 Left 🞏 Right 🞏 Both

🞏 Cyst aspiration/drainage 🞏 Left 🞏 Right 🞏 Both

🞏 Cyst ablation 🞏 Left 🞏 Right 🞏 Both

Tubal surgery 🞏 Fimbrioplasty 🞏 Left 🞏 Right 🞏 Both

🞏 Tuboplasty 🞏 Left 🞏 Right 🞏 Both

🞏 Lysis of adhesions (salpingolysis) 🞏 Left 🞏 Right 🞏 Both

🞏 Salpingectomy 🞏 Left 🞏 Right 🞏 Both

Peritoneum surgery 🞏 Destruction of endometriosis, specify:

🞏 Electrosurgery (monopolar) 🞏 Electrosurgery (bipolar) 🞏 Laser type: __________

🞏 Other: __________

🞏 Excision of endometriosis, specify:

🞏 Scissors 🞏 Harmonic scalpel 🞏 Laser type: __________

🞏 Other: __________

Number of specimens: ______ 🞏 Other ___________

Peritoneal fluid volume: __ __ __ ml Peritoneal Fluid: 🞏 Clear 🞏 Bloody

Bladder surgery Viscera entered 🞏 **No** 🞏 **Yes** 🞏 Specify: ______________________________________________

Ureter surgery 🞏 **No** 🞏 **Yes**

🞏 Left 🞏 Right 🞏 Both

🞏 Ureterolysis left

Mucosa entered 🞏 **No** 🞏 **Yes** 🞏 Specify: _________________________________

🞏 Primary repair 🞏 Segmental resection 🞏 Psoas hitch Specify: __________________

🞏 Ureterolysis right

Mucosa entered 🞏 **No** 🞏 **Yes** 🞏 Specify: _________________________________

🞏 Primary repair 🞏 Segmental resection 🞏 Psoas hitch Specify: __________________

Bowel surgery 🞏 **No** 🞏 **Yes**

Mucosa entered 🞏 **No** 🞏 **Yes** Specify:____________________________________________

🞏 Nodule removed

🞏 Discectomy

🞏 Bowel resection

🞏 Appendectomy

🞏 Other: _____________________________

Uterine surgery 🞏 **No** 🞏 **Yes**

🞏 Hysterectomy

🞏 Total 🞏 Subtotal 🞏 LAVH

🞏 Other _____________________

🞏 Myomectomy

Other procedures ___________ ____________ ___________ ____________ ____________ ____________

**VI**. **At conclusion of surgery:** Residual peritoneal endometriosis? **🞏 No** **🞏 Yes** **🞏** Location(s) _________ __________

Residual adhesions? **🞏 No** **🞏 Yes** **🞏** Location(s) _________ __________

Residual endometriomas? **🞏 No** **🞏 Yes** **🞏** Location(s) _________ __________

Residual nodules? **🞏 No** **🞏 Yes** **🞏** Location(s) _________ _________

**VII. Intraoperative complications**: **🞏** **No** **🞏 Yes**

**🞏** Type(s): ______________ ______________ ______________

**🞏** Treatment(s): ______________ ______________ ______________

**VIII. Any pathology observed during surgery:** **🞏** **No** **🞏** **Yes** 🡺 **If no: end of questionnaire**

**Visual diagnosis of endometriosis:** **🞏** **No** **🞏** **Yes** 🡺 **If no: go to question XII**

**🞏**Peritoneal

**🞏**Ovarian

**🞏**Deeply infiltrative

| **Perito-neum** | **Endometriosis** | **<1cm** | **1-3cm** | | **>3cm** |
| --- | --- | --- | --- | --- | --- |
|  | superficial | 1 **🞏** | 2 **🞏** | | 4 **🞏** |
|  | deep | 2 **🞏** | 4 **🞏** | | 6 **🞏** |
| **ovary** | Left superficial | 1 **🞏** | 2 **🞏** | | 4 **🞏** |
|  | deep | 4 **🞏** | 16 **🞏** | | 20 **🞏** |
|  | Right superficial | 1 **🞏** | 2 **🞏** | | 4 **🞏** |
|  | deep | 4 **🞏** | 16 **🞏** | | 20 **🞏** |
|  | **Pouch of Douglas obliteration** | **Partial** | | **Complete** | |
|  |  | 4 **🞏** | | 40 **🞏** | |
| **ovary** | **Adhesions** | **<1/3 enclosure** | **1/3 – 2/3** | | **>2/3 enclosure** |
|  | Left filmy | 1 **🞏** | 2 **🞏** | | 4 **🞏** |
|  | dense | 4 **🞏** | 8 **🞏** | | 16 **🞏** |
|  | Right filmy | 1 **🞏** | 2 **🞏** | | 4 **🞏** |
|  | dense | 4 **🞏** | 8 **🞏** | | 16 **🞏** |
| **tube** | Left filmy | 1 **🞏** | 2 **🞏** | | 4 **🞏** |
|  | dense | 4 **🞏** * | 8 **🞏 *** | | 16 **🞏** |
|  | Right filmy | 1 **🞏** | 2 **🞏** | | 4 **🞏** |
|  | dense | 4 **🞏** * | 8 **🞏 *** | | 16 **🞏** |

Revised American Fertility Society Score

** If the fimbriated end of the fallopian tube is completely enclosed, change the point assignment to 16*

Mark the total area of endometriosis, possibly of multiple lesions, NOT just the largest lesion

**Deeply infiltrative endometriosis (DIE) 🞏** **No** **🞏** **Yes**

**🞏** Pelvic side wall **🞏** Left **🞏** Right

**🞏** Ureter **🞏** Left **🞏** Right

**🞏** Posterior Cul-de-sac (Pouch of Douglas)

**🞏** Rectum

**🞏** Sigmoid

**🞏** Bladder

**🞏** Parametrium

**🞏** Uterosacral ligament **🞏** Left **🞏** Right

**🞏** Vagina

**🞏** Other ____________________________

**IX. Location of endometriosis, number and appearance of lesions:**

**LEFT SIDE**

| **Location of**  **Endometriosis** |  | **Appearance and Number of Lesions/Adhesions*** | | | | | | | | **Location of**  **the sample collected^§^** |
| --- | --- | --- | --- | --- | --- | --- | --- | --- | --- | --- |
|  | **Clear**  **A** | **Red**  **B** | **White**  **C** | **Blue/Black**  **D** | **BrownE** | **VascularF** | **Filmy Adhesion G** | **Dense Adhesion H** | **Control**  **Biopsy** |  |
| Left pelvic sidewall **🞏** |  |  |  |  |  |  |  |  |  | **🞏** _________ |
| Left utero-sacral ligament **🞏** |  |  |  |  |  |  |  |  |  | **🞏** _________ |
| Left ovary – serosa **🞏** |  |  |  |  |  |  |  |  |  | **🞏** _________ |
| Left tube – serosa **🞏** |  |  |  |  |  |  |  |  |  | **🞏** _________ |
| Others **🞏** __ __ __ __ __ __ |  |  |  |  |  |  |  |  |  | **🞏** _________ |

**RIGHT SIDE**

| **Location of**  **Endometriosis** |  | **Appearance and Number of Lesions/Adhesions*** | | | | | | | | **Location of**  **the sample collected^§^** |
| --- | --- | --- | --- | --- | --- | --- | --- | --- | --- | --- |
|  | **Clear**  **A** | **Red**  **B** | **White**  **C** | **Blue/Black**  **D** | **BrownE** | **VascularF** | **Filmy Adhesion G** | **Dense Adhesion H** | **Control**  **Biopsy** |  |
| Right pelvic sidewall **🞏** |  |  |  |  |  |  |  |  |  | **🞏** _________ |
| Right utero-sacral ligament **🞏** |  |  |  |  |  |  |  |  |  | **🞏** _________ |
| Right ovary – serosa **🞏** |  |  |  |  |  |  |  |  |  | **🞏** _________ |
| Right tube – serosa **🞏** |  |  |  |  |  |  |  |  |  | **🞏** _________ |
| Others **🞏** __ __ __ __ __ __ |  |  |  |  |  |  |  |  |  | **🞏** _________ |

**CENTRAL AREA**

| **Location of**  **Endometriosis** |  | **Appearance and Number of Lesions/Adhesions*** | | | | | | | | **Location of**  **the sample collected^§^** |
| --- | --- | --- | --- | --- | --- | --- | --- | --- | --- | --- |
|  | **Clear**  **A** | **Red**  **B** | **White**  **C** | **Blue/Black**  **D** | **BrownE** | **VascularF** | **Filmy Adhesion G** | **Dense Adhesion H** | **Control**  **Biopsy** |  |
| Uterovesical pouch/  Anterior cul-de-sac **🞏** |  |  |  |  |  |  |  |  |  | **🞏** _________ |
| Pouch of Douglas/  Posterior cul-de-sac **🞏** |  |  |  |  |  |  |  |  |  | **🞏** _________ |
| Uterus – serosa **🞏** |  |  |  |  |  |  |  |  |  | **🞏** _________ |
| Bladder – deep infiltrating **🞏** |  |  |  |  |  |  |  |  |  | **🞏** _________ |
| Bladder – serosa **🞏** |  |  |  |  |  |  |  |  |  | **🞏** _________ |
| Colon – deep infiltrating **🞏** |  |  |  |  |  |  |  |  |  | **🞏** _________ |
| Colon – serosa **🞏** |  |  |  |  |  |  |  |  |  | **🞏** _________ |
| Vagina **🞏** |  |  |  |  |  |  |  |  |  | **🞏** _________ |
| Others **🞏** __ __ __ __ __ __ |  |  |  |  |  |  |  |  |  | **🞏** _________ |

* Check multiple options if mixed colour; insert the number of lesions seen

**^§^** Please insert the appearance and number of lesion(s) the sample is collected from (A-H)

Peritoneal pouches/pockets **🞏** **No** **🞏** **Yes**

Location(s): ____________ ____________ ____________

Depth: ______________ Diameter: ___________________

Diaphragm **🞏** **No** **🞏** **Yes**

**🞏** Left Describe: _______________________________________________________

**🞏** Right Describe: _______________________________________________________

Control biopsy taken: **🞏** **No** **🞏** **Yes**

Location(s): 1. ________________ 2. _________________ 3. ____________________

**X. Endometrioma:** **🞏** **No** **🞏** **Yes**

**🞏** Left size(s): 1. __ __cm 2. __ __cm 3. __ __cm

**🞏** Right size(s): 1. __ __cm 2. __ __cm 3. __ __cm

**🞏** Sent to histology

🞏 Sample collected for research: 🞏 Left 🞏 Right

**XI. Endometriotic nodule:** Pouch of Douglas **🞏** **No** **🞏** **Yes** 🞏 Size* __ __ X __ __ X__ __ cm __ __ X __ __ X__ __ cm

Vagina **🞏** **No** **🞏** **Yes** 🞏 Size* __ __ X __ __ X__ __ cm __ __ X __ __ X__ __ cm

Bladder **🞏** **No** **🞏** **Yes** 🞏 Size* __ __ X __ __ X__ __ cm __ __ X __ __ X__ __ cm

Appendix **🞏** **No** **🞏** **Yes** 🞏 Size* __ __ X __ __ X__ __ cm __ __ X __ __ X__ __ cm

Small bowel **🞏** **No** **🞏** **Yes** 🞏 Size* __ __ X __ __ X__ __ cm __ __ X __ __ X__ __ cm

Sigmoid colon **🞏** **No** **🞏** **Yes** 🞏 Size* __ __ X __ __ X__ __ cm __ __ X __ __ X__ __ cm

Rectum **🞏** **No** **🞏** **Yes** 🞏 Size* __ __ X __ __ X__ __ cm __ __ X __ __ X__ __ cm

🞏 Location: _____________________ ____________________

🞏 Full thickness: **🞏** **No** **🞏** **Yes** **🞏** **No** **🞏** **Yes**

🞏 Distance from anus (bowel nodule): ______cm ______cm

* Clinical estimate

**XII. Additional findings:**

Fibroids (Myoma) **🞏** **No** **🞏** **Yes**

**🞏** Submucous **🞏** Number __ __ __ **🞏** Size* __ __ cm __ __ cm

**🞏** Size* __ __ cm __ __ cm

**🞏** Intramural **🞏** Number __ __ __ **🞏** Size* __ __ cm __ __ cm

**🞏** Size* __ __ cm __ __ cm

**🞏** Subserous **🞏** Number __ __ __ **🞏** Size* __ __ cm __ __ cm

**🞏** Size* __ __ cm __ __ cm

* Clinical estimate

Adhesions (w/o evidence of endometriosis) **🞏** **No** **🞏** **Yes** **🞏** Location(s) ___________________________________________

**🞏** Filmy

**🞏** Dense

**🞏** Co-apted

**🞏** Obstruction

Congenital anomaly **🞏** **No** **🞏** **Yes** 🡺 If yes, type(s) ____________________ ____________________

Non-endometriotic ovarian cyst **🞏** **No** **🞏 Yes** 🡺 If yes, side: **🞏** Left Suspected type ____________________

**🞏** Right Suspected type ____________________

Any other findings __________________________________________________________________________________________

Procedure was: **🞏** more complex/difficult than expected

🞏 as complex/difficult as expected

🞏 less complicated/difficult than expected

**XIII. Endometriosis Fertility Index (EFI):**
