# Supplementary material for: World Endometriosis Research Foundation Endometriosis Phenome and Biobanking Harmonisation Project: I. Surgical phenotype data collection in endometriosis research
Source: Fertil Steril. 2014 Nov;102(5):1213–22. doi: 10.1016/j.fertnstert.2014.07.709 (PMC4230690; doi:10.1016/j.fertnstert.2014.07.709)
Supplement: Supplemental Appendix 2 — Minimum (required) surgical form (EPHect MSF). [file mmc2.docx]

Surgeon ID: ________________________ Patient ID: ________________________ Date: __ __ /__ __ /__ __ __ __

DD MM YYYY

**I. Menses:** LMP: __ __ /__ __ /__ __ __ __ Cycle day: __ __ Currently bleeding? **🞏** **No** **🞏** **Yes**

DD MM YYYY

**II. Current hormonal treatment: 🞏** **No** **🞏** Do not know **🞏** **Yes**

**🞏** COCP **🞏** POP **🞏** Depot progestin

**🞏** GnRH agonist **🞏** GnRH antagonist **🞏** IUCD

**🞏** Other ___________________

Last application: __ __ /__ __ /__ __ __ __

DD MM YYYY

**III. Previous surgical diagnosis of endometriosis:** **🞏** **No** **🞏** Do not know **🞏** **Yes**

**IV.** **Current surgery:** Procedure(s): __________________________________________________________________________

**V. Any pathology observed during surgery:** **🞏** **No** **🞏** **Yes** 🡺 **If no: end of questionnaire**

**Visual diagnosis of endometriosis:** **🞏** **No** **🞏** **Yes** 🡺 **If no: go to question VII**

| **Perito-neum** | **Endometriosis** | **<1cm** | **1-3cm** | | **>3cm** |
| --- | --- | --- | --- | --- | --- |
|  | superficial | 1 **🞏** | 2 **🞏** | | 4 **🞏** |
|  | deep | 2 **🞏** | 4 **🞏** | | 6 **🞏** |
| **ovary** | Left superficial | 1 **🞏** | 2 **🞏** | | 4 **🞏** |
|  | deep | 4 **🞏** | 16 **🞏** | | 20 **🞏** |
|  | Right superficial | 1 **🞏** | 2 **🞏** | | 4 **🞏** |
|  | deep | 4 **🞏** | 16 **🞏** | | 20 **🞏** |
|  | **Pouch of Douglas obliteration** | **Partial** | | **Complete** | |
|  |  | 4 **🞏** | | 40 **🞏** | |
| **ovary** | **Adhesions** | **<1/3 enclosure** | **1/3 – 2/3** | | **>2/3 enclosure** |
|  | Left filmy | 1 **🞏** | 2 **🞏** | | 4 **🞏** |
|  | dense | 4 **🞏** | 8 **🞏** | | 16 **🞏** |
|  | Right filmy | 1 **🞏** | 2 **🞏** | | 4 **🞏** |
|  | dense | 4 **🞏** | 8 **🞏** | | 16 **🞏** |
| **tube** | Left filmy | 1 **🞏** | 2 **🞏** | | 4 **🞏** |
|  | dense | 4 **🞏** * | 8 **🞏 *** | | 16 **🞏** |
|  | Right filmy | 1 **🞏** | 2 **🞏** | | 4 **🞏** |
|  | dense | 4 **🞏** * | 8 **🞏 *** | | 16 **🞏** |

Revised American Fertility Society Score

** If the fimbriated end of the fallopian tube is completely enclosed, change the point assignment to 16*

Mark the total area of endometriosis, possibly of multiple lesions, NOT just the largest lesion

**VI. Location of endometriosis, number and appearance of lesions:**

**LEFT SIDE**

| **Location of**  **Endometriosis** |  | **Appearance and Number of Lesions/Adhesions*** | | | | | | | | **Location of**  **the sample collected^§^** |
| --- | --- | --- | --- | --- | --- | --- | --- | --- | --- | --- |
|  | **Clear**  **A** | **Red**  **B** | **White**  **C** | **Blue/Black**  **D** | **BrownE** | **VascularF** | **Filmy Adhesion G** | **Dense Adhesion H** | **Control**  **Biopsy** |  |
| Left pelvic sidewall **🞏** |  |  |  |  |  |  |  |  |  | **🞏** _________ |
| Left utero-sacral ligament **🞏** |  |  |  |  |  |  |  |  |  | **🞏** _________ |
| Left ovary – serosa **🞏** |  |  |  |  |  |  |  |  |  | **🞏** _________ |
| Left tube – serosa **🞏** |  |  |  |  |  |  |  |  |  | **🞏** _________ |
| Others **🞏** __ __ __ __ __ __ |  |  |  |  |  |  |  |  |  | **🞏** _________ |

**Supplementary Appendix I (Continued)**

**RIGHT SIDE**

| **Location of**  **Endometriosis** |  | **Appearance and Number of Lesions/Adhesions*** | | | | | | | | **Location of**  **the sample collected^§^** |
| --- | --- | --- | --- | --- | --- | --- | --- | --- | --- | --- |
|  | **Clear**  **A** | **Red**  **B** | **White**  **C** | **Blue/Black**  **D** | **BrownE** | **VascularF** | **Filmy Adhesion G** | **Dense Adhesion H** | **Control**  **Biopsy** |  |
| Right pelvic sidewall **🞏** |  |  |  |  |  |  |  |  |  | **🞏** _________ |
| Right utero-sacral ligament **🞏** |  |  |  |  |  |  |  |  |  | **🞏** _________ |
| Right ovary – serosa **🞏** |  |  |  |  |  |  |  |  |  | **🞏** _________ |
| Right tube – serosa **🞏** |  |  |  |  |  |  |  |  |  | **🞏** _________ |
| Others **🞏** __ __ __ __ __ __ |  |  |  |  |  |  |  |  |  | **🞏** _________ |

**CENTRAL AREA**

| **Location of**  **Endometriosis** |  | **Appearance and Number of Lesions/Adhesions*** | | | | | | | | **Location of**  **the sample collected^§^** |
| --- | --- | --- | --- | --- | --- | --- | --- | --- | --- | --- |
|  | **Clear**  **A** | **Red**  **B** | **White**  **C** | **Blue/Black**  **D** | **BrownE** | **VascularF** | **Filmy Adhesion G** | **Dense Adhesion H** | **Control**  **Biopsy** |  |
| Uterovesical pouch/  Anterior cul-de-sac **🞏** |  |  |  |  |  |  |  |  |  | **🞏** _________ |
| Pouch of Douglas/  Posterior cul-de-sac **🞏** |  |  |  |  |  |  |  |  |  | **🞏** _________ |
| Uterus – serosa **🞏** |  |  |  |  |  |  |  |  |  | **🞏** _________ |
| Bladder – deep infiltrating **🞏** |  |  |  |  |  |  |  |  |  | **🞏** _________ |
| Bladder – serosa **🞏** |  |  |  |  |  |  |  |  |  | **🞏** _________ |
| Colon – deep infiltrating **🞏** |  |  |  |  |  |  |  |  |  | **🞏** _________ |
| Colon – serosa **🞏** |  |  |  |  |  |  |  |  |  | **🞏** _________ |
| Vagina **🞏** |  |  |  |  |  |  |  |  |  | **🞏** _________ |
| Others **🞏** __ __ __ __ __ __ |  |  |  |  |  |  |  |  |  | **🞏** _________ |

* Check multiple options if mixed colour; insert the number of lesions seen

**^§^** Please insert the appearance and number of lesion(s) the sample is collected from (A-G)

**VII. Endometrioma:** **🞏** **No** **🞏** **Yes**

**🞏** Left size(s): 1. __ __cm 2. __ __cm 3. __ __cm

**🞏** Right size(s): 1. __ __cm 2. __ __cm 3. __ __cm

**🞏** Sent to histology

🞏 Sample collected for research: 🞏 Left 🞏 Right

**VIII. Additional findings:**

Fibroids (Myoma) **🞏** **No** **🞏** **Yes**

Adhesions (w/o evidence of endometriosis) **🞏** **No** **🞏** **Yes**

Non-endometriotic ovarian cyst **🞏** **No** **🞏 Yes**

Any other findings __________________________________________________________________________________________
