# Supplementary material for: World Endometriosis Research Foundation Endometriosis Phenome and Biobanking Harmonisation Project: I. Surgical phenotype data collection in endometriosis research
Source: Fertil Steril. 2014 Nov;102(5):1213–22. doi: 10.1016/j.fertnstert.2014.07.709 (PMC4230690; doi:10.1016/j.fertnstert.2014.07.709)
Supplement: Supplemental Appendix 3 — Example pictures of endometriotic lesions. [file mmc3.docx]

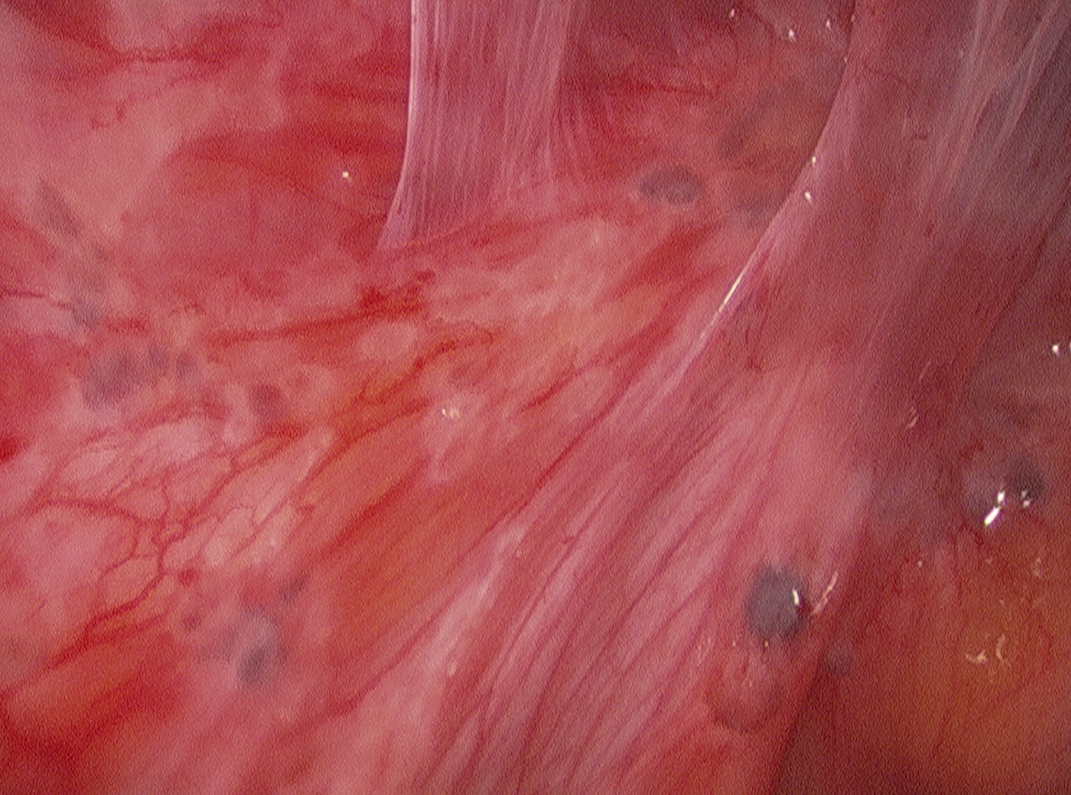

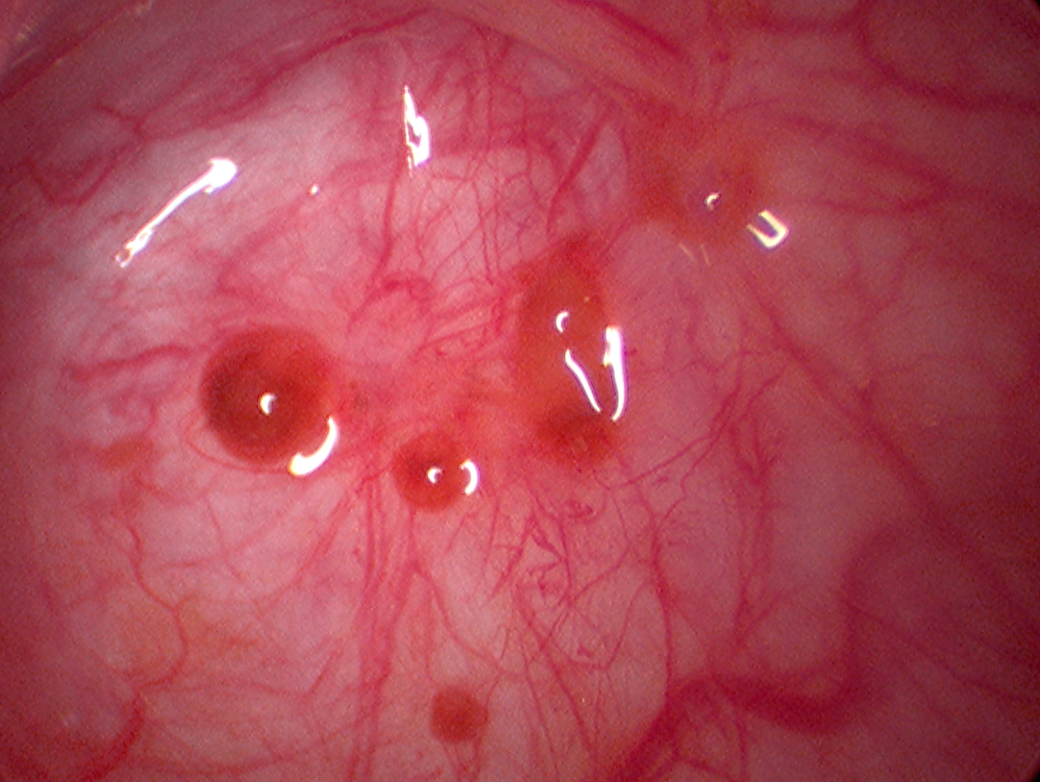


Dense adhesions/fibrosis (likely as result of

deep infiltrating lesion (bladder nodule))

Allen-Masters peritoneal window;

red/vascularized superficial peritoneal lesions

Red/brown superficial peritoneal lesions

Filmy adhesions; white and blue/black

superficial peritoneal lesions


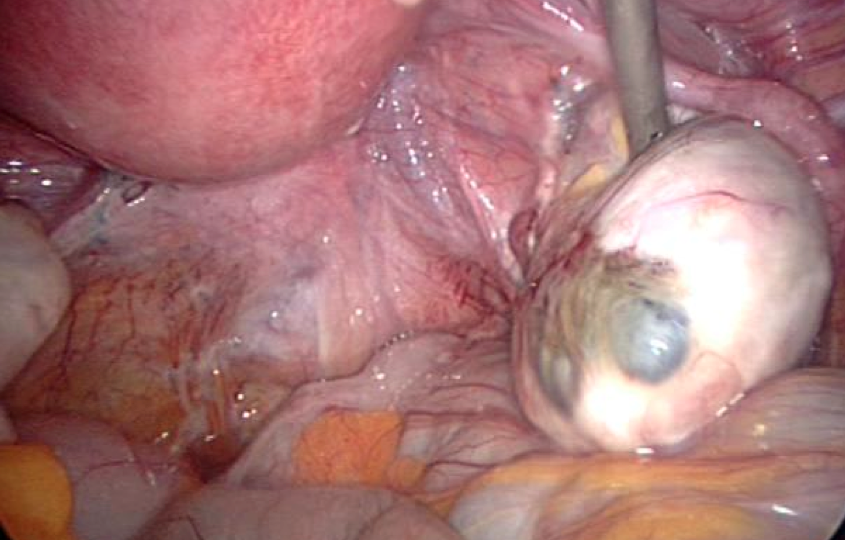

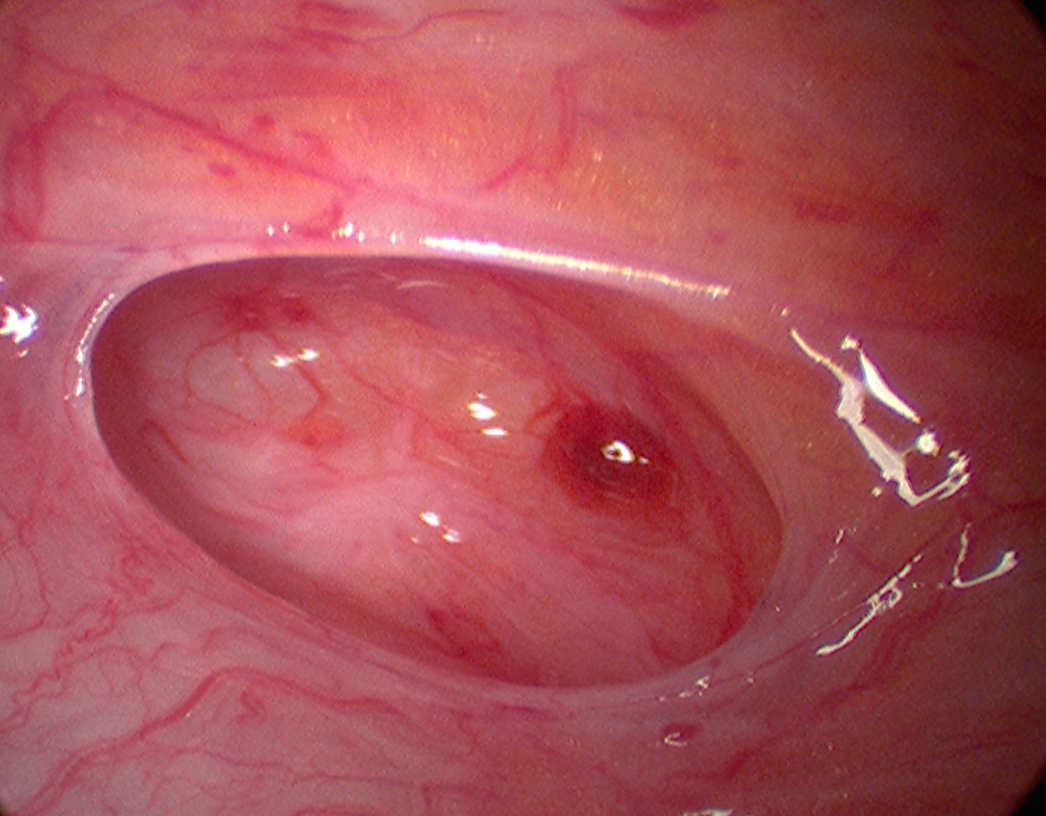


Superficial ovarian blue/black lesions fibrosis;

deep infiltrating lesions left utero-sacral

ligament


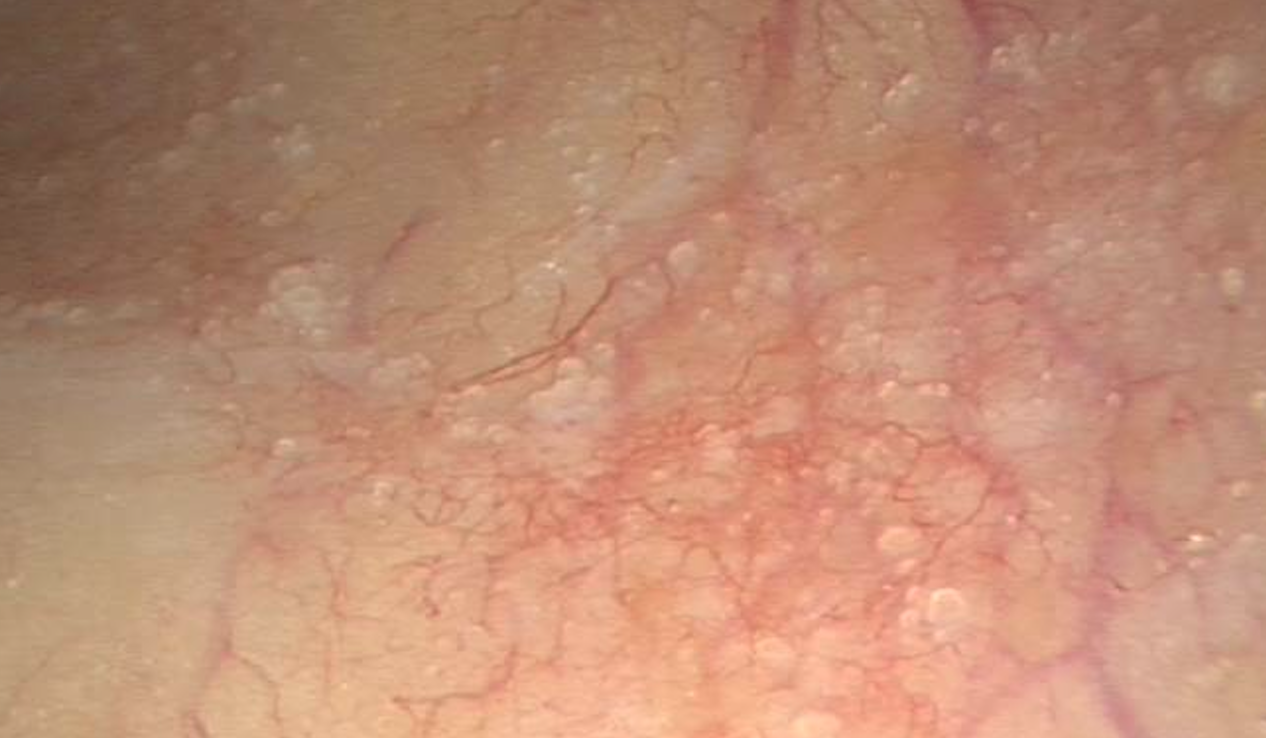

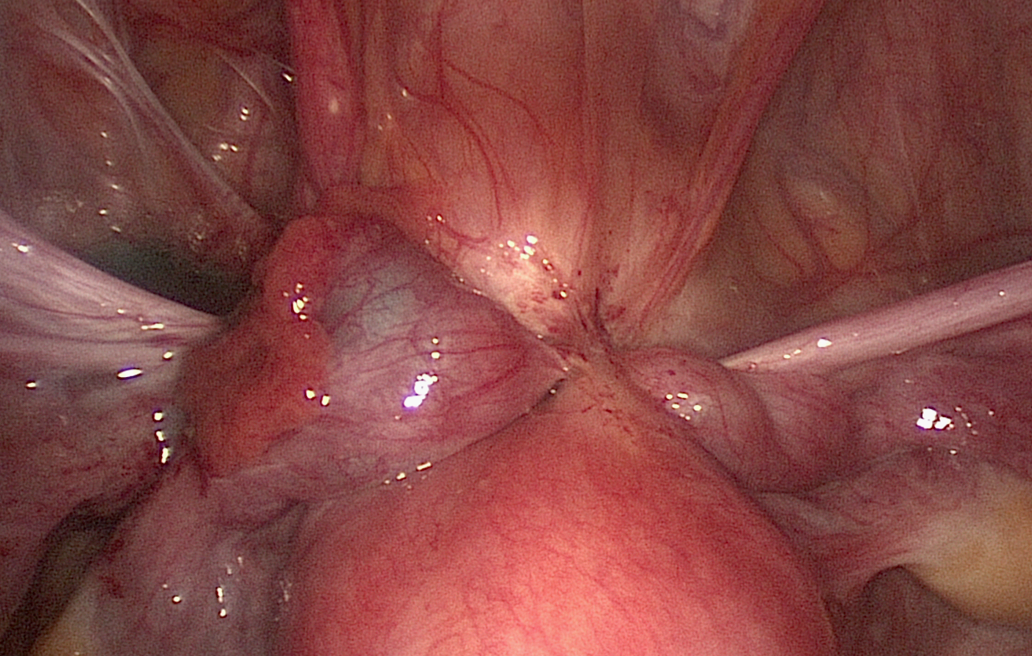


Superficial vesicular/vascular peritoneal

lesions
